# Avidity of Antibodies against HSV-2 and Risk to Neonatal Transmission among Mexican Pregnant Women

**DOI:** 10.1155/2013/140142

**Published:** 2013-08-06

**Authors:** Antonia Herrera-Ortiz, Carlos Jesús Conde-Glez, Dayana Nicté Vergara-Ortega, Santa García-Cisneros, Ma. Leonidez Olamendi-Portugal, Miguel Angel Sánchez-Alemán

**Affiliations:** ^1^Centro de Investigación sobre Enfermedades Infecciosas, Instituto Nacional de Salud Pública, Avenida Universidad No. 655, Colonia Santa María Ahuacatitlán, Cerrada Los Pinos y Caminera, CP 62100, Cuernavaca, Morelos, Mexico; ^2^Centro de Investigación en Salud Poblacional, Instituto Nacional de Salud Pública, Avenida Universidad No. 655, Colonia Santa María Ahuacatitlán, Cerrada Los Pinos y Caminera, CP 62100, Cuernavaca, Morelos, Mexico; ^3^Universidad Politécnica del Estado de Morelos, Boulevard Cuauhnáhuac No. 566, Colonia Lomas del Texcal, CP 62550, Jiutepec, Morelos, Mexico

## Abstract

*Objective*. To determine HSV-2 seroprevalence, risk factors, and antibody avidity among a sample of Mexican pregnant women. *Material and Methods*. The avidity test was standardized with different urea concentrations and incubation times; the cut-off point was calculated to determine the low avidity (early infection). IgG antibodies against HSV-2 were detected from pregnant and postpartum women from Morelos, Mexico, and the avidity test was performed to positive samples. Multivariate regression logistic analysis was employed to evaluate demographic and sexual behavior characteristics associated with HSV-2 infection. *Results*. HSV-2 seroprevalence among Mexican women analyzed was 14.5% (333/2300), demographic factors (location of General Hospital, age, education level, and civil status), and risky sexual behaviors (STI self-report and number of sexual partners during last year) were associated with HSV-2 infection. Seventeen women were detected with low avidity antibodies (early infection) with a cut-off point of 66.1%. *Conclusions*. HSV-2 infection was common among this group of women from Mexico; the avidity test detected women with recent infections, and these women were more likely to transmit HSV-2 to their neonates. Neonatal herpes has no epidemiological surveillance, the disease could be overlooked, and so more studies are needed to estimate the magnitude of neonatal infection.

## 1. Introduction

Herpes Simplex Virus (HSV) belongs to Herpesviridae family and has four basic structures: core with DNA double strand, icosahedral capsid, tegument, and lipidic envelope, and also HSV presents two basic properties, latency and neurovirulence. HSV-1 can cause oral lesions, and HSV-2 is the principal agent of genital herpes and could cause recurrent ulcers but is asymptomatic in 80% of cases [1, 2].

Genital herpes is a frequent infection during pregnancy, one-fifth of women have antibodies against HSV-2 [3], one-tenth of pregnant women infected with HSV-2 have genital viral shedding [4], and finally, 5% of women with genital viral shedding could transmit the infection to their neonates [5]. USA reported a neonatal herpes incidence of 31.25 for 100,000 newborns in Washington [5] and 13.3/100,000 in New York [6]. Vertical transmission leading to neonatal herpes virus infection may occur at vaginal delivery, which can cause congenital anomalies. Forty-five percent of neonatal herpes cause the localized form in mouth, eyes, or skin; 30% of cases arise nervous system infections, with lethargy, convulsions, and loss of appetite, with or without skin lesions; the remaining 25% of neonatal herpes are associated with disseminated infection (clinically not different from bacterial sepsis); this type of neonatal form can affect lung, liver, and brain; the lethality is around 80% [6–10].

HSV-2 serology among pregnant women detects seronegative women during early pregnancy for followup during pregnancy, because women with primary (early) infection during second half of pregnancy have a higher risk to transmit the virus to neonate [7]. It is not possible to classify the infection between primary or nonprimary by HSV-2 serology, because IgM antibodies can be present also during viral reactivation [11]. The detection of IgM antibodies against cytomegalovirus (another member of Herpesviridae that also causes neonatal infection) do not indicate primary infection, however, the IgG avidity test (force measurement of antigen-antibody interaction) can discriminate between early and late infections, and the low avidity is produced during the first month after infection and the high avidity, later [12]. The low avidity for HSV-2 has been associated with early infections and the high avidity with persistent infections [13]. The aims of the current study were to determine the HSV-2 seroprevalence and characteristics associated with infection among a sample of Mexican pregnant women, and also the IgG avidity index was evaluated to detect early HSV-2 infections.

## 2. Material and Methods

### 2.1. Standardization of Avidity Test

Consecutive samples from a cohort study to detect HSV-2 seroincidence were employed [13]. If both samples (with an interval of six months between them) were seropositive to HSV-2, the second sample was used to standardize the avidity test. We evaluated 43 serum samples at different urea concentrations and incubation times; the median (Me) and interquartile range were employed to describe the results. The ELISA commercial test (ELISA-Focus) was modified in the step after the serum sample incubation, the plate was washed, and we added an incubation time with urea. The cut-off point was calculated with a nonparametric analysis and confidence intervals at 95% [14].

### 2.2. Study Design and Statistical Analyses

A transversal study was carried out during 2006–2009 among pregnant and puerperal women to detect antibodies against *T. pallidum* [15]. The hospitals studied were General Hospital (GH) from Cuautla (Dr. Mauro Belauzarán Tapia), GH from Jojutla (Dr. Ernesto Meana San Román), GH from Axochiapan (Dr. Ángel Ventura Neri), and GH from Tetecala (Dr. Rodolfo Becerril de la Paz), all from Morelos, center of Mexico. The women signed an informed consent, answered a questionnaire about demographic characteristics, prenatal care, and sexual behaviors, and provided a blood sample to maternal syphilis detection. The serum was frozen at −20°C until detection of IgG antibodies against HSV-2, with a commercial ELISA test (ELISA-Focus). A simple frequency analysis was carried out to describe population characteristics, and the bivariate and multivariate logistic regression analyses were carried out to describe variables associated with HSV-2 infection, with backward steps methodology. We calculated crude and adjusted odds ratio with confidence intervals at 95% (CI_95%_), and the statistical analysis was performed with SPSS 15.0.

## 3. Results

**Figure 1:**
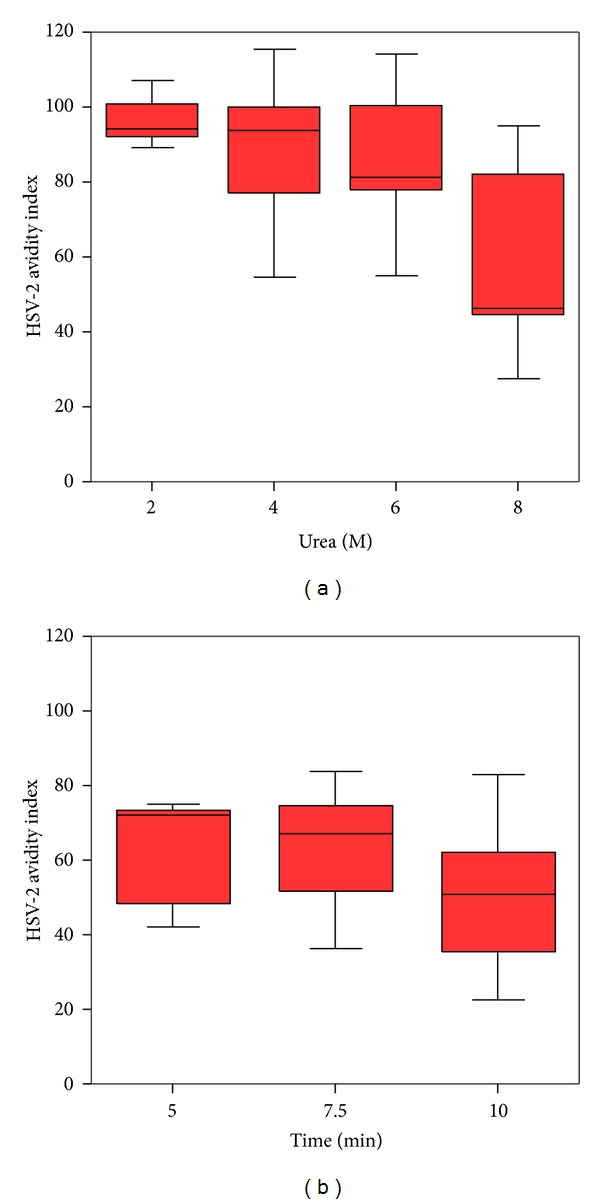
Standardization of avidity test to IgG antibodies against HSV-2. Molar urea concentration (a), time in minutes (b). Box plot. Median, 25, and 75 quartile, limits to outliers.

We evaluated four urea concentrations for the avidity test; the high avidity index was detected with 2 M and 4 M of urea, 95% and 94%, respectively, and low avidity (64%) with 8 M. Urea 6 M was used in current study because it showed an intermediate avidity index, 82% (Figure 1(a)). Three incubation times were evaluated, the avidity was 51% (IQR 34%) at 10 min, 71% (IQR 35%) at 7.5 min, and 72% (IQR 29%) at 5 min, and we employed five minutes because the avidity was similar to 7.5 min, but with less variation, 29% versus 35% (Figure 1(b)). Forty-three seropositive samples to HSV-2 were used to calculate the cut-off point. The median of avidity index was 85.4% (IQR 15%), the percentile 5% was 66.2%, and this was the cut-off point. We defined high avidity (late infection) if avidity index was equal or higher than 66.2% and low avidity (early infection) with avidity index equal or lower than 66.1%.

We tested 2300 serum samples of pregnant and puerperal women from Morelos, Mexico, half of women were between 21–30 years old, a third was married, and 4% was illiterate. One-third of the Mexican women tested reported condom use ever in life, 90% of pregnant women had one sexual partner during last year, and almost 20% reported infidelity from last sexual partner. Thirty percent of women mentioned four or less prenatal visits, and a quarter did not report urine exam an indirect variable of adequate prenatal care.  Table 1 shows demographic, antenatal care, and sexual behavior characteristics from the women analyzed. 

The overall prevalence of IgG antibodies against HSV-2 was 14.5% (333/2300, CI_95%_ 13.0–15.9), and the HSV-2 seroprevalence varied from 9.4% in Tetecala GH to 16.2% in Jojutla GH. There was a direct association between increasing age and increasing HSV-2 seroprevalence; from 8.6% among women 20 years old or younger to 26.4% among women 31 years or older, a third of divorced women had HSV-2 infection, similar to women without schooling. The pregnant women with three or more sexual partners during last year and women that reported infidelity of partner had 31.1% and 17.8% of HSV-2 seroprevalence, respectively. The logistic regression analysis showed that pregnant women 31 years or older, (OR_A_ = 4.3), pregnant divorced women (OR_A_ = 4.0), and pregnant women without schooling (OR_A_ = 3.1) had a higher risk to HSV-2 infection. Pregnant women with history of STI had 1.7 times more risk to HSV-2 infection, and women with three or more sexual partners during last year showed twice the risk to HSV-2 infection. None of the prenatal care variables were associated with HSV-2 infection.  Table 2 presents the logistic regression of all the variables analyzed.

**Figure 2:**
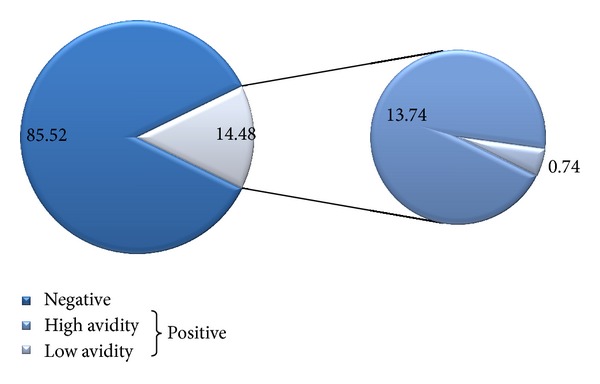
HSV-2 seroprevalence and avidity level among puerperal and pregnant women from Morelos, Mexico.

The avidity test was performed to 333 samples of women with IgG antibodies against HSV-2; 17 samples showed a low avidity index, median 59.0% (IQR 15.5%), and the remaining samples had high avidity, median 86.7% (IQR 13.0%). The low avidity was detected in 0.74% (CI_95%_ 0.39–1.01%) from the total of pregnant women analyzed (*n* = 2, 300), but among women with HSV-2 antibodies (*n* = 333), 5.11% (CI_95%_ 2.73–7.48%) had low avidity (early infection).  Figure 2 shows HSV-2 seroprevalence and avidity index from Mexican pregnant women analyzed.

## 4. Discussion

The avidity test was standardized with serum samples from one Mexican population, with a cut-off point of 62.2%. Ashley-Morrow [16] calculated a cut-off of 40%, and they detected 80% of early infections (6 weeks or less) with this cut-off point. The difference between both cut-off points could be because of the different populations analyzed or because the chemical agents for the avidity test were different (urea 6 M versus NaSCN 0.75 M). 

The HSV-2 seroprevalence among pregnant women from Morelos, Mexico, was 14.5%, lower than 20.7% obtained from National Health and Nutrition Survey 2000 from Mexico [17]; the difference could be due to the age of participants, because a third of population of National Survey was 50 years or older. Infection with HSV-2 among a sample of Mexican pregnant women was lower than USA (22%) [3] and Switzerland (21.2%) [18] but higher than Italy (7.6–8.4%) [19] or India (8.7%) [20]. The HSV-2 seroprevalence showed differences among the General Hospitals analyzed; GH of Tetecala had the lowest seroprevalence; all the localities from this municipality had less than 5,000 habitants, different to Axochiapan (48.0%), Jojutla (34.2%), and Cuautla (11.9%) [21]; HSV-2 infection could be more prevalent among urban than rural communities, like syphilis infection that was more prevalent among urban locations [15]. Older pregnant women (≥31 years old) had more risk to HSV-2 infection, because of their higher exposure time to the virus [22] and because IgG antibodies remain lifelong. Pregnant women with lower education level had a higher HSV-2 risk, because a lower education level is associated with risky sexual behaviors [23]. A higher number of sexual partners increase the probability to encounter with an infected partner; accordingly the studied Mexican women from Morelos with three or more sexual partners had twice the risk to HSV-2 infection.

Using data about HSV-2 seroprevalence, genital viral shedding, and neonatal HSV-2 infection from USA, we calculated for this study that from 1000 pregnant women, 145 women would have antibodies against HSV-2, 15 pregnant women present genital viral shedding, and 0.75 women could transmit the infection to newborn, with a rate of neonatal herpes of 0.75 case per 1000 newborns (75 cases/100,000 newborns), higher than reported in Washington, USA (31.25/100,000). There is no information about neonatal herpes in Mexico, because is not obligatory to report it; however, the National Center for Epidemiological Surveillance and Diseases Control (CENAVECE) [24] has reported genital herpes cases among children younger than 1 year (70 cases between years 2000–2010), with rates between 0 and 1.06 cases/100,000 persons. 

## 5. Conclusions

The HSV-2 seroprevalence among a sample of pregnant and puerperal women from Mexico was 14.5%; also 0.74% of women had low avidity IgG antibodies to HSV-2, marker of early infections; these 17 women had a high probability to present genital viral shedding and could transmit HSV-2 to their newborns. The current study did not evaluate viral genital shedding among pregnant women; however, the frequency of women with low avidity index and the high HSV-2 seroprevalence detected, point out the necessity to know the importance of neonatal herpes in Mexico, to implement detection national programs, like those directed to HIV or syphilis.

## Figures and Tables

**Table 1:** Demographic, sexual behaviors, and antenatal care characteristics of puerperal and pregnant women from Morelos, Mexico.

Variables	*n*	%
General Hospital		
Cuautla	1006	43.7
Jojutla	690	30.0
Axochiapan	349	15.2
Tetecala	255	11.1
Age		
≥31 years old	390	17.0
21–30 years old	1163	50.6
≤20 years old	747	32.5
Marital status		
Separated	32	1.4
Single	276	12.0
Law marriage	1224	53.2
Married	768	33.4
Education level		
Nothing	91	4.0
Elementary school	752	32.7
Junior high	1016	44.2
High school or upper	441	19.2
Lifetime condom use		
Yes	678	29.5
No	1622	70.5
History of STI		
Yes/not known	99	4.3
No	2201	95.7
Partners during last year		
Three or more	45	2.0
Two	176	7.7
One	2079	90.4
Partner infidelity		
Yes	455	19.8
Not known	667	29.0
No	1178	51.2
Prenatal consultations		
0–2	404	17.6
3–4	315	13.7
5 or more	1581	68.7
Person in charge of pregnancy control		
No one	127	5.5
Midwife	43	1.9
Nursery	76	3.3
Doctor	2054	89.3
Urine exam		
No	555	24.1
Yes	1745	75.9

**Table 2:** HSV-2 seroprevalence and risk factors among puerperal and pregnant women from Morelos, Mexico.

Variable	% HSV-2	OR_C_ (CI_95%_)	OR_A_ (CI_95%_)
General Hospital			
Cuautla	14.8	1.7 (1.1–2.6)^&^	1.6 (1.0–2.7)^&^
Jojutla	16.2	1.9 (1.2–3.0)^&^	1.9 (1.2–3.2)^&^
Axochiapan	13.8	1.5 (0.9–2.6)	1.4 (0.8–2.5)
Tetecala	9.4	1.0	1.0
Age			
≥31 years old	26.4	3.8 (2.7–5.4)^&^	4.3 (3.0–6.1)^&^
21–30 years old	14.3	1.8 (1.3–2.4)^&^	1.9 (1.4–2.6)^&^
≤20 years old	8.6	1.0	1.0
Marital status			
Separated	31.3	3.8 (1.7–8.3)^&^	4.0 (1.8–9.0)^&^
Single	18.1	1.9 (1.3–2.7)^&^	2.2 (1.5–3.3)^&^
Law marriage	15.6	1.5 (1.2–2.0)^&^	1.8 (1.4–2.4)^&^
Married	10.7	1.0	1.0
Education level			
Nothing	29.7	4.1 (2.4–7.2)^&^	3.1 (1.8–5.6)^&^
Elementary school	18.0	2.1 (1.5–3.1)^&^	2.0 (1.3–2.9)^&^
Junior high	12.8	1.4 (0.9–2.1)^&^	1.5 (1.0–2.1)^&^
High school or upper	9.3	1.0	1.0
Lifetime condom use			
Yes	14.1	0.9 (0.7–1.2)	
No	15.3	1.0	
History of STI			
Yes/not known	21.2	1.6 (0.99–2.7)	1.7 (1.0–3.0)^&^
No	14.2	1.0	1.0
Partners during last year			
Three or more	31.1	2.9 (1.5–5.6)^&^	2.2 (1.1–4.4)^&^
Two	23.9	2.0 (1.4–2.9)^&^	1.6 (1.1–2.4)^&^
One	13.3	1.0	1.0
Partner infidelity			
Yes	17.8	1.6 (1.2–2.2)^&^	
Not known	16.8	1.5 (1.1–2.0)^&^	
No	11.9	1.0	
Prenatal consultations			
0–2	19.1	1.5 (1.2–2.1)^&^	
3-4	15.2	1.2 (0.8–1.67)	
5 or more	13.2	1.0	
Person in charge of pregnancy control			
No one	18.9	1.4 (0.9–2.2)	
Midwife	23.3	1.8 (0.9–3.8)	
Nursery	11.8	0.8 (0.4–1.7)	
Doctor	14.1	1.0	
Urine exam			
No	18.4	1.5 (1.1–1.9)	
Yes	13.2	1.0	

OR_C_: crude odds ratio; OR_A_: adjusted odds ratio; ^&^statistical significance.

## References

[1] Whitley RJ, Roizman B (2001). Herpes simplex virus infections. *The Lancet*.

[2] Wald A, Zeh J, Selke S (2000). Reactivation of genital herpes simplex virus type 2 infection in asymptomatic seropositive persons. *The New England Journal of Medicine*.

[3] Brown ZA, Gardella C, Wald A, Morrow RA, Corey L (2005). Genital herpes complicating pregnancy. *Obstetrics and Gynecology*.

[4] Andrews WW, Kimberlin DF, Whitley R, Cliver S, Ramsey PS, Deeter R (2006). Valacyclovir therapy to reduce recurrent genital herpes in pregnant women. *The American Journal of Obstetrics and Gynecology*.

[5] Brown ZA, Wald A, Morrow RA, Selke S, Zeh J, Corey L (2003). Effect of serologic status and cesarean delivery on transmission rates of herpes simplex virus from mother to infant. *The Journal of the American Medical Association*.

[6] Rudnick CM, Hoekzema GS (2002). Neonatal herpes simplex virus infections. *The American Family Physician*.

[7] Sauerbrei A, Wutzler P (2007). Herpes simplex and varicella-zoster virus infections during pregnancy: current concepts of prevention, diagnosis and therapy—part 1: herpes simplex virus infections. *Medical Microbiology and Immunology*.

[8] Kimberlin DW, Lin CY, Jacobs RF (2001). Natural history of neonatal herpes simplex virus infections in the acyclovir era. *Pediatrics*.

[9] Corey L, Wald A (2009). Maternal and neonatal herpes simplex virus infections. *The New England Journal of Medicine*.

[10] Anzivino E, Fioriti D, Mischitelli M (2009). Herpes simplex virus infection in pregnancy and in neonate: status of art of epidemiology, diagnosis, therapy and prevention. *Virology Journal*.

[11] Ashley RL (1998). Type specific antibodies to HSV-1 and -2: review of methodology. *Herpes*.

[12] Revello MG, Gerna G (2002). Diagnosis and management of human cytomegalovirus infection in the mother, fetus, and newborn infant. *Clinical Microbiology Reviews*.

[13] Yáñez-Alvarez I, Martínez-Salazar MF, Conde-González CJ, García-Serrato AB, Sánchez-Alemán MA (2011). Seroprevalencia y seroincidencia del virus del herpes simple tipo 2 en personas que viven con VIH. *Enfermedades Infecciosas y Microbiología *.

[14] Solberg HE (2004). The IFCC recommendation on estimation of reference intervals. The RefVal program. *Clinical Chemistry and Laboratory Medicine*.

[15] Yáñez-Alvarez I, Conde-González CJ, Uribe-Salas FJ, Olamendi-Portugal ML, García-Cisneros S, Sánchez-Alemán MA (2012). Maternal/child seroprevalence of antibodies against *Treponema pallidum* at four general hospitals in the state of Morelos, Mexico. *Archives of Medical Research*.

[16] Morrow RA, Friedrich D, Krantz E, Wald A (2004). Development and use of a type-specific antibody avidity test based on herpes simplex virus type 2 glycoprotein G. *Sexually Transmitted Diseases*.

[17] Uribe-Salas F, Palma-Coca O, Sánchez-Alemán MA, Olamendi M, Juárez-Figueroa L, Conde-Glez CJ (2009). Population-based prevalence of antibodies against herpes simplex virus type 2 and socio-demographic characteristics in Mexico. *Transactions of the Royal Society of Tropical Medicine and Hygiene*.

[18] Kucera P, Gerber S, Marques-Vidal P, Meylan PRA (2012). Seroepidemiology of herpes simplex virus type 1 and 2 in pregnant women in Switzerland: an obstetric clinic based study. *European Journal of Obstetrics Gynecology and Reproductive Biology*.

[19] Suligoi B, Cusan M, Santopadre P (2000). HSV-2 specific seroprevalence among various populations in Rome, Italy. *Sexually Transmitted Infections*.

[20] Biswas D, Borkakoty B, Mahanta J (2011). Seroprevalence and risk factors of herpes simplex virus type-2 infection among pregnant women in Northeast India. *BMC Infectious Diseases*.

[21] Mundo F Consejo estatal de población. http://www.coespomor.gob.mx/investigacion_poblacion/marginacion/1_marginacion.pdf.

[22] Uribe-Salas F, Hernández-Avila M, Juárez-Figueroa L, Conde-Glez CJ, Uribe-Zúñiga P (1999). Risk factors for herpes simplex virus type 2 infection among female commercial sex workers in Mexico City. *International Journal of STD and AIDS*.

[23] Kirby D (2002). The impact of schools and school programs upon adolescent sexual behavior. *Journal of Sex Research*.

[24] Anuarios de morbilidad. http://www.dgepi.salud.gob.mx/anuario/html/anuarios.html.

